# Hook, Line, and Sinker: Anatomical Distribution of Head and Neck Fishing Injuries in Children

**DOI:** 10.7759/cureus.110197

**Published:** 2026-06-03

**Authors:** Beatrice R Bacon, Matthaeus Hendricks, Ryan Dewan, Michele M Carr

**Affiliations:** 1 Otolaryngology-Head and Neck Surgery, University of Pittsburgh School of Medicine, Buffalo, USA; 2 Otolaryngology, Jacobs School of Medicine and Biomedical Sciences, University at Buffalo, Buffalo, USA

**Keywords:** children, fishhook, fishing, foreign body, laceration

## Abstract

Introduction

Fishhooks are designed to grab tissue. The goal of this study was to determine which head and neck locations were most involved in pediatric fishing-related injuries.

Methods

The National Electronic Injury Surveillance System (NEISS) was queried for fishing-related head and neck injuries between 2013 and 2022 in patients <19 years old. Demographics, including age, gender, and race, were collected. Cause of injury, head and neck location, and disposition were analyzed. Rashes (N=57) and non-fishing injuries (N=30) were excluded.

Results

A total of 697 children, 546 (78.3%) male and 151 (21.7%) female, were included. Mean age was 9.5 (95% CI 9.1-9.8) years. Causes of injury included fishhooks (N=590, 84.6%), falls (N=52, 7.5%), fishing poles or nets (N=31, 4.4%), sinkers (N=13, 1.9%), fish (N=2, 0.3%), and other causes (N=9, 1.25%). The national estimate for pediatric head and neck fishing-related injuries was 37,467 for this 10-year period.

There was a significant difference in the anatomical distribution of hook vs. non-hook injuries (p<0.001). Fishhook injuries mostly occurred in the face (N=237/590, 40.2% of fishhook injuries); non-hook injuries were most common on the head (N=48/107, 44.9% of non-hook injuries). Fishhook injuries were more common than non-hook-related injuries in the ear (N=80/590, 13.6% vs. N=1/107, 0.9%) and the eyelids (N=47/590, 8.0% vs. N=4/107, 3.7%).

Children with pole-related injuries (N=31) were significantly younger (mean 6.5 vs. 9.6 years; p<0.001); these were most common in the face (N=15/31, 48.4%) and mouth (N=10/31, 32.3%).

Conclusion

Pediatric fishhook impalements in the face and ear are common, requiring otolaryngologists to be adept in their management.

## Introduction

Fishing is one of the most popular outdoor recreational activities for children and adolescents in the United States. According to data from the Outdoor Foundation and the Recreational Boating and Fishing Foundation, 7.8 million children aged 6 to 12 years in the United States fished at least once in 2022, equating to a 27% national participation rate for the age group [1]. Similarly, 4.9 million adolescents aged 13 to 17 years fished at least once in 2022, equating to a 23% national participation rate [1]. Rates of participation among these age groups are increasing; annual participation steadily increased 2% and 6% among children and adolescents, respectively, from 2019 to 2021 [1].

With increasing participation, there is heightened concern for fishing-related injuries, particularly of the head and neck region. Behind swimming, fishing is the second-most common aquatic recreational activity leading to emergency department presentation for treatment of injury [2]. Simply, fishhooks are designed to grab tissue. There are numerous different shapes of fishhooks, some of which are designed to be more aggressive and thus are more likely to cause injury. A fishhook is characterized by its number of tips, presence of a barb, and relation of its barb to its shank [3]. One of the most common fishhooks used among freshwater anglers is a barbed hook with a parallel, J-shaped tip, appropriately named the J hook [3]. The parallel tip increases the depth of penetration, while the barb prevents removal. While the design of the J hook increases fishing success rate, it also poses greater risk for injury to the user and complicates removal. Further, this risk is increased through the use of treble hooks, which are composed of three barbed J hooks fused together [3]. Another popular type of fishhook, the circle hook, has a tip bent perpendicular to its shank and often catches fish in the corner of the mouth, requiring less time to unhook the fish compared to J hooks [3]. While circle hooks, particularly barbless circle hooks, may not be as effective as a fishing tactic, the use of this type of fishhook comes with tremendous benefits for wildlife conservation and human safety [3]. While the popularity of barbed hooks is a key factor in the prevalence of fishing-related injuries, fishhooks are not the only source of injury among young anglers. Falls, fishing poles, nets, sinkers, and even fish themselves have the potential to cause injury.

In their 2018 retrospective review of the National Electronic Injury Surveillance System (NEISS) database, Vajdic et al. found that pediatric fishing-related injuries were most commonly caused by hooks and lures, and were most likely to occur on the arm and hand [4]. The goal of our study was to determine which locations within the head and neck were most likely to be involved in pediatric fishing-related injuries.

## Materials and methods

Experimental design

This retrospective study was reviewed by the Institutional Review Board for the State University of New York (SUNY) at Buffalo. This project was deemed non-human research. A query was conducted using the NEISS database, operated by the United States Consumer Product Safety Commission, between the years 2013 and 2022. Children under 19 years old who suffered various injuries to the head and neck while fishing were identified. Definitions for each variable are available for reference in the 2021 NEISS Coding Manual [5].

Study population

The NEISS Estimates Query Builder was used to search for “Fishing (activity/apparel/equipment; excluding fishing knives)” (code 3223). The maximum age was set to 19 years. Under the “Body Part” heading, selections were made for “head” (code 75), “face (including eyelid, eye area, and nose)” (code 76), “eyeball” (code 77), “mouth (including lips, tongue, and teeth)” (code 88), “neck” (code 89), and “ear” (code 94). NEISS assigns a single body-part code to each injury record; therefore, injuries were classified into one anatomical category only, and no records were coded in multiple body-part categories. Each NEISS record represented a single injury event and served as the unit of analysis. Under the “Diagnosis” search criteria, all injuries were initially included. Case narratives were manually reviewed to exclude dermatitis/rash presentations unrelated to traumatic fishing injuries (N=57) and injuries not directly attributable to fishing activities or equipment (N=30). The “Disposition” search criteria included all available options. Those not admitted to the hospital were classified as “no injury” (code 0), “treated and released or examined and released without treatment” (code 1), or “left without being seen or left against medical advice (AMA)” (code 6), while those admitted were classified as “treated and transferred to another hospital” (code 2), “treated and admitted for hospitalization within the same facility” (code 4), or “held for observation” (code 5).

The output search criteria were grouped by year of injury, age in years, sex, race, injury type, and final disposition. Data on ethnicity were not collected prior to 2019, and there are gaps in this information prior to this time.

Statistical analysis

Quantitative data analyses were conducted using IBM SPSS Statistics for Windows, Version 27 (Released 2019; IBM Corp., Armonk, New York, United States). Continuous variables were analyzed using independent samples t-tests with Welch’s correction for unequal variances, and categorical variables were analyzed using chi-square tests. Test statistics (t and χ^2^), degrees of freedom, and corresponding p-values are reported, with p<0.05 considered statistically significant.

## Results

Incidence and demographics

From 2013 to 2022, there were 697 entries in the NEISS database of patients younger than 19 years who sustained fishing-related injuries to the head or neck. The national estimate for pediatric head and neck fishing-related injuries is 37,467 for this 10-year period. National estimates were generated using the statistical weights assigned to each NEISS case by the Consumer Product Safety Commission, allowing extrapolation from participating emergency departments to national injury estimates.

The mean patient age at time of injury was 9.5 years (95% CI 9.1-9.8), and the majority of children were male (N=546, 78.3%). Patient race and ethnicity were not reliably recorded throughout our study period, and were therefore left out of this analysis.

Mechanisms and locations of injury

Injuries were most commonly caused by fishhooks (N=590, 84.6%) and falls (N=52, 7.5%), followed by fishing poles or nets (N=31, 4.4%), sinkers (N=13, 1.9%), fish (N=2, 0.3%), and other causes (N=9, 1.3%).

**Figure 1:**
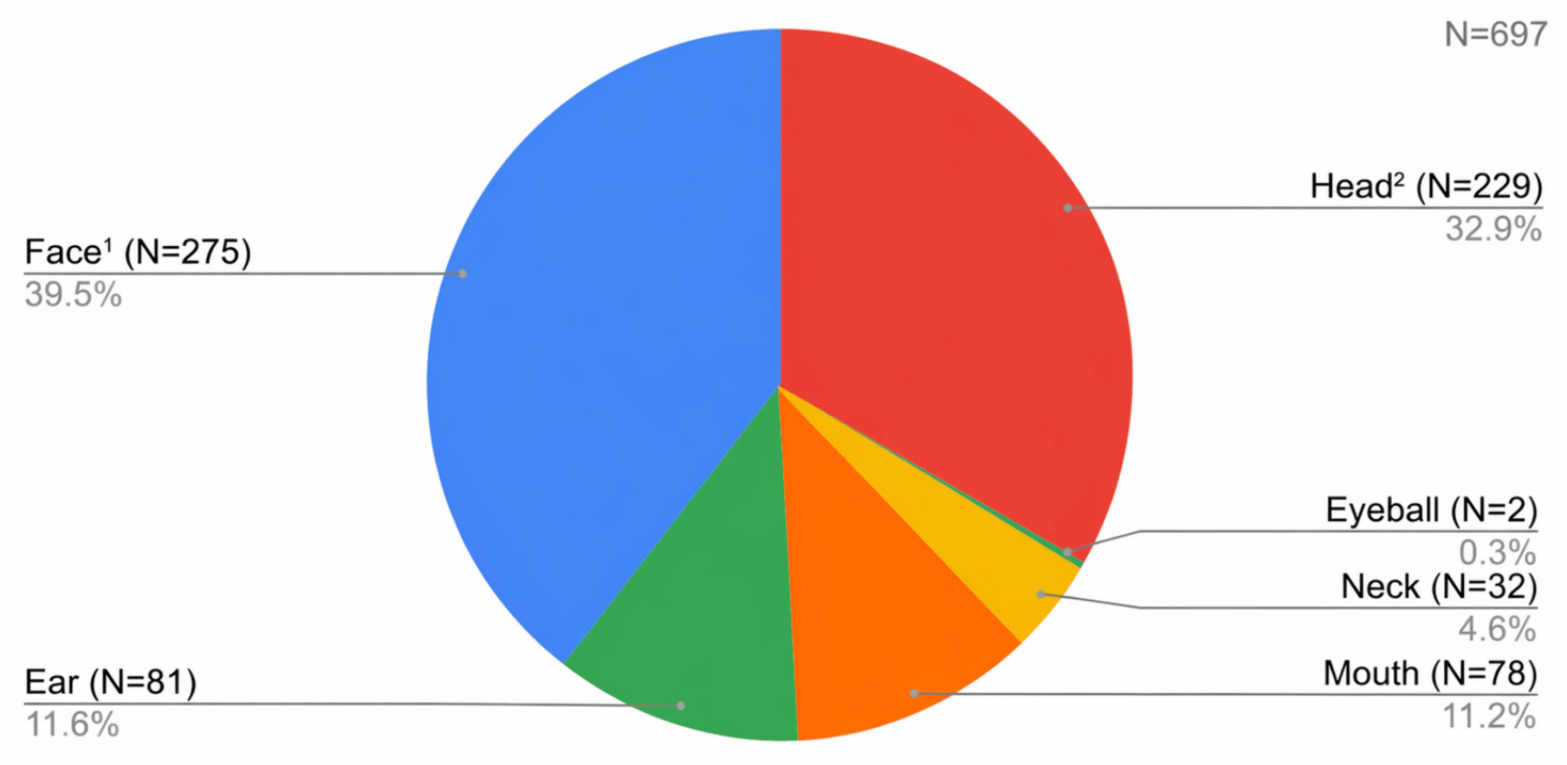
Locations of fishing-related injuries in children ^1^ including the eyelid, eye area, and nose; ^2^ including lips, tongue, and teeth. Injuries predominantly occurred on the face and head, while ear, mouth, neck, and eyeball injuries were less frequent.

Injuries most commonly occurred on the face (N=275, 39.5%) and head (N=229, 32.9%), shown in Figure 1. The most common diagnosis at the time of presentation was foreign body (N=511, 73.3%), followed by laceration (N=77, 11.0%), puncture (N=48, 6.9%), internal organ injury (N=20, 2.9%), and contusion or abrasion (N=17, 2.4%). Less common diagnoses included concussion (N=5), dental injury (N=3), fracture (N=2), thermal burn (N=1), hematoma (N=1), strain or sprain (N=1), and dermatitis or conjunctivitis (N=1).

There was a significant difference in the anatomical distribution of fishhook versus non-fishhook injuries (χ^2^(5)=29.62, p<0.001). Fishhook injuries were most common in the face (N=237/590, 40.1%), while non-fishhook injuries were most common on the head (N=48/107, 44.9%). Fishhook injuries were more common than non-fishhook-related injuries in the ear (N=80/590, 13.6% vs. N=1/107, 0.9%) and the eyelids (N=47/590, 8.0% vs. N=4/107, 3.7%), as seen in Table 1.

**Table 1:** Distribution of fishhook vs. non-fishhook injuries by body part ^1^ including the eyelid, eye area, and nose; ^2^ including lips, tongue, and teeth. Chi-square test was used to compare anatomical distribution between groups (χ^2^(5)=29.62, p<0.001).

Body Part	Fishhook, N (%), N=590	Non-fishhook, N (%), N=107
Face^1^	237 (40.2)	38 (35.5)
Head	181 (30.7)	48 (44.9)
Mouth^2^	63 (10.7)	15 (14.0)
Neck	27 (4.6)	5 (4.7)
Eyeball	2 (0.3)	0 (0)
Ear	80 (13.6)	1 (0.9)

Children with pole/net-related injuries (N=31) were significantly younger than those with other fishing-related injuries (N=666) (6.54±4.07 vs. 9.59±4.59 years; Welch’s t(33.65)=-4.05, p<0.001). When fishing pole-related injuries did occur, they were most common in the face (N=15/31, 48.4%) and mouth (N=10/31, 32.3%).

## Discussion

Common fishing-related diagnoses

Fishing tackle includes a variety of objects suited to assist an angler with catching fish. This may include fishhooks, lines, bait, lures, fishing rods, sinkers, nets, spears, traps, and many other pieces of equipment, which can result in unique mechanisms of injury. In our study, most children who presented to emergency departments with fishing-related injuries were diagnosed with foreign body (N=511, 73.3%), laceration (N=77, 11.0%), or puncture (N=48, 6.9%). These findings are similar to those of Hahn et al., who found that foreign body was the most common diagnosis in adult recreational or competitive anglers presenting to emergency departments in Australia, followed by cuts or lacerations and puncture wounds [6]. In another study, open wounds were the most common fishing-related diagnosis in children and adults (34%), followed by foreign bodies in soft tissue (19%), usually caused by fishhooks [2].

Fishhooks

Fishhooks typically cause superficial soft tissue injury to the hand, face, head, or upper extremity [7]. The linear forces caused by the motion of fishing drive the point of the fishhook parallel to the skin, preventing penetration into deeper tissue. In this study, most injuries were caused by fishhooks, and the face was the most common location of fishhook injury within the head and neck. This finding is consistent with a previous study conducted in Newfoundland, which examined adult fishhook-related emergency department presentations between 2013 and 2015 [8]. Another retrospective study of 37 patients at the KK Women’s and Children’s Hospital emergency department in Singapore found fishhook injuries of the eyelid were slightly more prevalent than those of the rest of the face (19.8% vs. 8.1%) [9]. These findings were not replicated in our study; only 16.3% of all facial injuries were fishhook-related eyelid injuries (N=45/275) compared to 70.0% of all facial injuries caused by fishhooks affecting other parts of the face (N=192/275). Regardless, both studies show that fishhook injury to the finger is more common than any location within the head and neck [8,9].

Injury evaluation

Once hooked, there are a variety of methods that experienced fishermen will often attempt before presenting to a clinical setting, the most common of which is the string-yank method. This method requires wrapping the fishing line around the bend of the fishhook, depressing the eye of the hook, and firmly pulling the string in a rapid motion [10-12]. If field attempts remain unsuccessful, the patient may require an emergency department evaluation to determine fishhook type, anatomic location, and best removal technique. It is crucial to obtain a thorough history, including a timeline of events, attempts at removal before arrival, and tetanus status [13]. During the patient’s physical examination, an initial assessment of any critical areas of injury, including the neck, airway, eyes, eyelids, arteries, and genitalia, is recommended, as injury to these locations may warrant immediate consultation [14]. An assessment of neurologic and vascular status proximal and distal to the wound is also recommended prior to any intervention, including the administration of local anesthetic [13]. The decision to order radiographic studies depends on the anatomic location and depth of injury. If the entire fishhook is visible, a clinical diagnosis can be made without imaging, protecting the patient from unnecessary radiation and conserving healthcare resources [8,13].

Other causes of fishing-related injuries

Most fishing-related injuries are caused by cutting or piercing objects, followed by low falls, weights or sinkers, and animal-related injuries [2]. In our study, the vast majority of fishing-related injuries were caused by fishhooks, as is supported by the existing literature; only 107 patients (15.4%) experienced injuries not caused by fishhooks. Depending on the velocity and force of movement, sinkers can act similarly to low-velocity bullets [15]. In one case report, a 38-year-old man was admitted to the hospital with a sinker embedded above his left eye [15]. The patient did not have any intracranial pathology or neurologic deficits, and the sinker was removed via external ethmoidectomy [15]. Two patients (0.3%) in our study were injured by fish. One patient was a seven-year-old boy who was hit in the cheek by a fin, and the other was an 11-year-old boy who accidentally caught a shark and was poked in the eye while trying to release it. In the literature, fish-related injuries were typically presented as case reports and were most commonly caused by spikes [2].

Safety recommendations

Safety practices can help to reduce fishhook injuries among pediatric patients. Children are not required to be accompanied by an adult while fishing; if an adult is present, he or she is required to have a fishing license only if actively participating [16]. Most states do not require a child under the age of 16 years to have a fishing license if he or she has proof of age [16]. Regardless of age, children must follow local regulations regarding bag limits, methods, and seasonal regulations for each species, and parents must take responsibility for teaching their children proper safety techniques and providing appropriate supervision while fishing [16].

There are numerous pieces of safety equipment available for use across all ages to reduce the incidence of fishing-related injuries. Wrap-around eyewear with coverage against UVA and UVB is recommended to protect against accidents during line casting and against branches if fishing near trees [17-19]. The addition of polarized, plastic polycarbonate lenses can further help to reduce eye strain and aid in fish visualization while removing the danger of glass lenses [18,20,21].

Regarding fishing equipment, the use of barbless and circle hooks has been found to reduce the likelihood of angler injury while also reducing post-release fish mortality, providing conservation benefits [3,20]. Children’s fishing rods, which are sized appropriately for different age groups, are encouraged as they are easier to control and minimize risk of injury to any nearby bystanders [20]. A waterproof fishing first aid kit, containing a hook remover and basic wound care supplies, can prove helpful in situations in which immediate medical attention is required [19]. Finally, to encourage safety practices during fishing, it may be helpful for merchants to distribute safety information to their young customers. Alternatively, this information could be distributed at the state level to fishermen requesting licenses and boater registrations [22].

Limitations

Our study has several inherent limitations related to the use of the NEISS database. The records within the database contain limited information about injury mechanism and circumstance. For example, an injury classified as a “fall” could be due to anything from syncope to slipping on a rock. There is no individual, analyzable data regarding the type of fishing or fishing instrumentation causing trauma, nor its severity and treatment. Fishermen often attempt fishhook removal in the field; therefore, there is the possibility that our study population represents a higher severity of injury compared to what exists in the general population. Additionally, the approximately 100 emergency departments that contribute to the NEISS database are selected as a “probability sample,” which aims to represent the general population. Hospitals can refuse to participate, affecting the generalizability of this study’s findings and potentially introducing selection bias. The Consumer Product Safety Commission provides resources to hospitals to encourage participation in the NEISS database, including payment for computers, training, and each case submitted. These incentives could potentially influence a hospital’s decision of whether to contribute to the database.

## Conclusions

This study highlights the significant prevalence of pediatric fishing-related injuries, particularly those involving fishhooks, with facial and ear regions being the most commonly affected. Our data suggest that pole-related injuries occur in younger children (mean age 6.5 years) and most commonly involve the face and mouth. Understanding these patterns can aid in injury prevention strategies and inform targeted safety measures to reduce the occurrence of head and neck injuries in children engaging in fishing activities.
